# Evaluation of the clinical efficacy of *Pediococcus acidilactici* CCFM6432 in alleviating depression

**DOI:** 10.20517/mrr.2024.33

**Published:** 2024-09-18

**Authors:** Peijun Tian, Hongyu Yang, Feng Hang, Gang Wang, Xuhua Mao, Xing Jin, Jianxin Zhao

**Affiliations:** ^1^State Key Laboratory of Food Science and Resources, Jiangnan University, Wuxi 214122, Jiangsu, China.; ^2^School of Food Science and Technology, Jiangnan University, Wuxi 214122, Jiangsu, China.; ^3^(Yangzhou) Institute of Food Biotechnology, Jiangnan University, Yangzhou 225004, Jiangsu, China.; ^4^National Engineering Research Center for Functional Food, Jiangnan University, Wuxi 214122, Jiangsu, China.; ^5^Yixing People’s Hospital Affiliated Jiangsu University, Yixing 214200, Jiangsu, China.

**Keywords:** *Pediococcus acidilactici*, depression, gut microbiota, lactic acid, immune

## Abstract

**Aim:** Accumulating evidence highlights the crucial role of the “gut-brain axis” and emphasizes the potential of dietary interventions to improve brain health through this pathway. This study assesses the effects of the probiotic *Pediococcus acidilactici* CCFM6432 on mood, sleep, and gastrointestinal function in patients with depressive disorder.

**Methods:** This clinical trial is a randomized, placebo-controlled study (Registration: ChiCTR2300071025). It enrolled 39 adult patients diagnosed with depressive disorder, who were randomly assigned to either the placebo control group (*n* = 19) or the CCFM6432 intervention group (*n* = 20). The intervention period spanned four weeks. Assessments were conducted at both the beginning and end of the trial, including comprehensive questionnaire evaluations and the collection of serum and fecal samples.

**Results:** In comparison to the placebo, treatment with CCFM6432 significantly decreased depression and anxiety scores, as well as ameliorated gastrointestinal dysfunction and poor sleep quality commonly associated with mood disorders. Microbiota analysis revealed an increase in species richness without notable changes in overall diversity, yet *Pediococcus* species was found to be more abundant post-treatment. Functional analysis indicated reduced activity in the NOD-like receptor signaling pathway, suggesting anti-inflammatory effects induced by the probiotic. Metabolomic profiling identified elevated levels of fecal lactic acid, which correlated with lower Hospital Anxiety and Depression Scale (HADS) scores, thereby linking probiotic metabolism to mood enhancement.

**Conclusion:** These findings imply that CCFM6432 may improve brain function by modulating gut microbiota and their mediated immune homeostasis, underscoring its potential as an adjunctive treatment for mental disorders.

## INTRODUCTION

Depression, as a psychiatric disorder with complex etiology, has garnered widespread attention globally. According to projections by the World Health Organization, depression is expected to become the leading cause of global disease burden by 2030^[1]^. Particularly in the post-pandemic era, there has been a significant increase in the risk of mood disorders worldwide^[2]^. However, due to the intricate pathogenesis of depression and substantial individual variations, the selection and efficacy of antidepressant medications are currently greatly constrained^[3,4]^. Drugs widely used in clinical practice, such as selective serotonin reuptake inhibitors and serotonin-norepinephrine reuptake inhibitors, often yield remission rates of less than 50%^[5]^.

In recent years, with the continuous expansion of knowledge related to the “gut-brain axis”, the correlation and even causality between changes in gut microbiota and mental health status have been gradually established^[6]^. The gut microbiota can participate in or promote the synthesis of various neurotransmitters, such as acetylcholine, γ-aminobutyric acid, and glutamate, thereby influencing the host’s emotions, memory, and cognitive function^[7]^. Compared to healthy individuals, patients with depression exhibit significant differences in gut microbial composition and often experience various gastrointestinal dysfunctions^[8-10]^. Transplanting fecal microbiota from patients with depression into the intestines of healthy mice can induce depressive-like behavior in the mice^[11]^. Conversely, transplanting gut microbiota from healthy volunteers into the intestines of patients with anxiety or depression has been shown to significantly reduce the patients’ anxiety and depression scores^[12,13]^. However, fecal microbiota transplantation faces significant potential safety risks, and the lack of donor screening and recipient adaptability assessment methods limits its widespread use in the treatment of depression^[14]^.

Nevertheless, this has not deterred researchers from exploring alternative approaches to harnessing the gut microbiota to improve emotional well-being. A plethora of preclinical studies suggest that the ingestion of probiotics can inhibit the occurrence of organismal inflammation and cortisol levels, reduce stress responses, enhance memory, and decrease the occurrence of host mood disorder-related symptoms^[10,15,16]^. These beneficial live organisms to host mental health are defined as “Psychobiotics”^[17]^. However, the positive effects of psychobiotics on depressive symptoms have shown inconsistent results in clinical trials. A systematic review based on 13 clinical trials revealed that while the results of seven trials (including reports from three independent cohorts of the same strain) confirmed a significant reduction in depression scores during probiotic supplementation, the remaining six trials did not yield beneficial outcomes^[18]^. This inconsistency is believed to be due to significant variations among different strains. Therefore, elucidating the material basis and mechanisms by which specific effective strains alleviate depression will greatly promote the selection and application of psychobiotics.

In this context, our previous research has demonstrated that continuous intake of a composite probiotic formulation containing *Pediococcus acidilactici* CCFM6432 can improve symptoms in patients with severe depression^[19]^. Furthermore, we confirmed through a chronic stress animal model that intervention with the single strain CCFM6432 can alleviate anxiety-like behaviors in mice. The aim of this study is to comprehensively evaluate the adjunctive therapeutic effect of *Pediococcus acidilactici* CCFM6432 on depressive symptoms through a randomized, placebo-controlled clinical trial and to explore the potential material basis for its efficacy.

## METHODS

### Probiotic strain and placebo

*Pediococcus acidilactici* CCFM6432 was obtained from the Culture Collection of Food Microorganisms (CCFM) at Jiangnan University. The probiotic formulation used in this trial consisted of freeze-dried powder of CCFM6432 and maltodextrin. The specific preparation process is as follows: After two successive activations on MRS solid medium (37 °C, cultured for 36-48 h), a single colony was picked and inoculated into MRS liquid medium. After incubating at 37 °C for 18 h, the culture was further propagated at the same conditions with a 1% (v/v) inoculum to increase biomass. A 2% (v/v) inoculum was then fermented in a 20-liter fermenter for 18 h. The fermentation broth was centrifuged to obtain bacterial slurry. Subsequently, the bacterial slurry was mixed with protectants at a ratio of 1:5 (w/v) (protectants composed of 13% skim milk, 3% sucrose, and 3% inulin) and freeze-dried to obtain the probiotic powder of CCFM6432. The probiotic powder was then packed into sachets (2 g/sachet, with a viable count of ≥ 10^9^ CFU/sachet). Maltodextrin placebo was prepared in the same manner without the addition of bacterial powder.

### Clinical trial design

This clinical trial is a randomized, placebo-controlled study. The study was conducted collaboratively by the Food Biotechnology Center of Jiangnan University and Yixing People’s Hospital. The clinical trial design complies with the Helsinki Declaration and has been approved by the Medical Ethics Committee of Yixing People’s Hospital (WXMHCIRB2023LLky002, Yixing, China). Clinical Trial Registration Number: ChiCTR2300071025.

In this study, we used G Power software to calculate the required sample size for participant recruitment, using the methodology from a previous study by Majeed *et al.* as a reference^[20]^. Specifically, in their study on the effects of probiotics on alleviating depression, the Hospital Anxiety and Depression Scale (HADS) scores were 13.60 ± 4.41 (Mean ± SD) before treatment and 5.90 ± 4.88 (Mean ± SD) after treatment. Using an alpha level of 0.05, a power (1-β) of 0.80, and an effect size of 0.94, we estimated the minimum total sample size to be 38 participants, with an actual power of 0.80. Consequently, at least 19 patients were required per group.

Inclusion criteria: (1) Clinical diagnosis of depressive disorder or anxiety disorder; (2) HADS score > 11; (3) Aged 18 years and above; (4) Primary school education or above, without speech expression disorders; (5) Subject or guardian has signed an informed consent form; (6) Subject fills out a written informed consent form, basic information questionnaire, dietary structure and lifestyle questionnaire, and commits to cooperate with the study throughout the entire trial period. Exclusion criteria: (1) Treatment-resistant depression; (2) Meets DSM-IV Axis I diagnosis of other mental disorders; (3) Suicidal or self-harming behavior; (4) Patients with schizophrenia, bipolar disorder, neurodegenerative diseases; (5) Severe physical illnesses such as HIV, history of epilepsy, history of heart disease, hyperthyroidism, and other serious physical illnesses; (6) Pregnant or lactating women; men or women during preconception (conception); (7) Use of antibiotics, probiotics, and their products in the past month.

**Figure 1:**
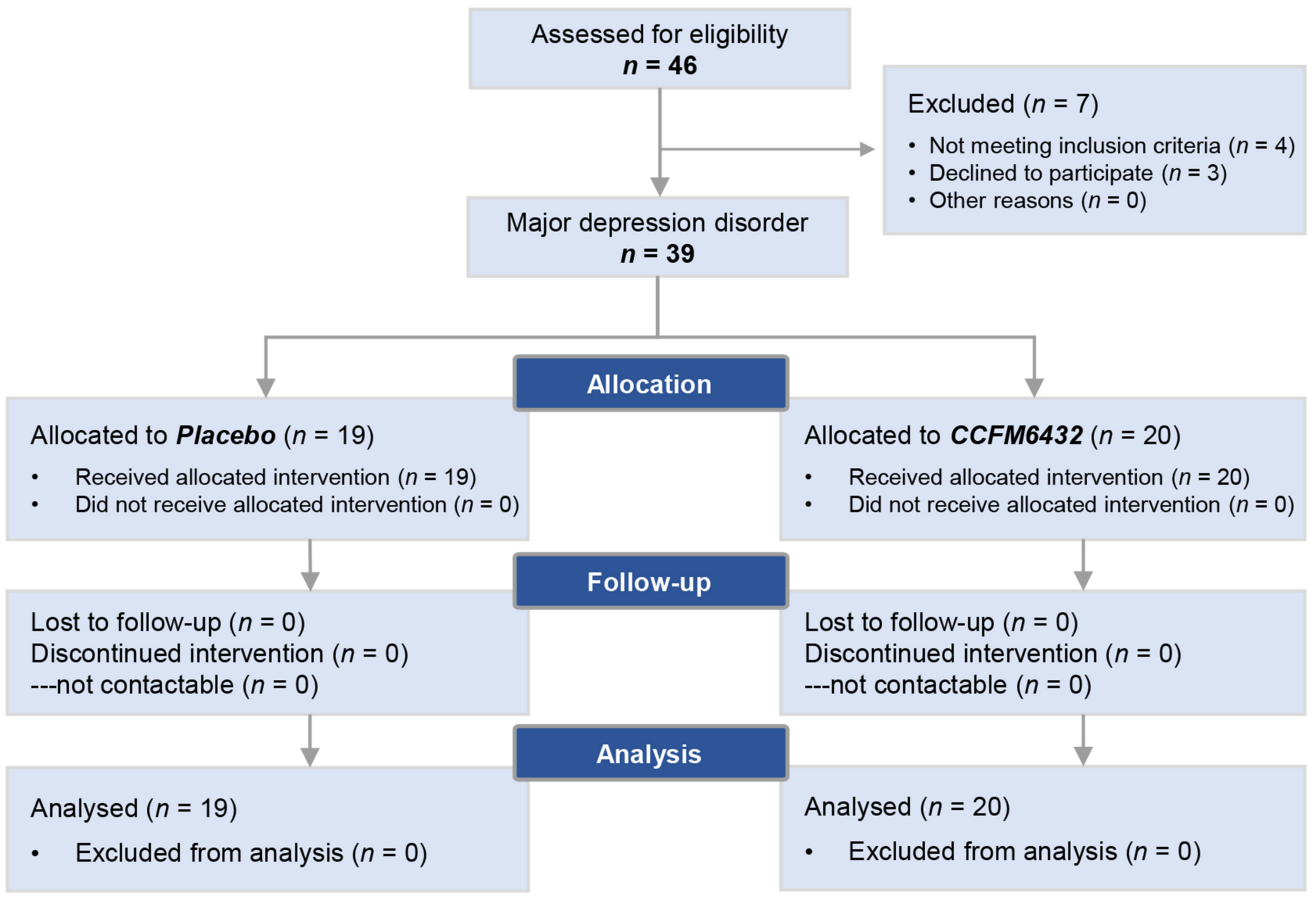
Flow chart of study procedures. A total of 46 patients were initially screened for this study. Thirty-nine patients who met the inclusion criteria were randomly assigned to either the Placebo group (*n* = 19) or the CCFM6432 group (*n* = 20). All participants completed the trial and were included in the data analysis.

The entire intervention period lasted for four weeks. Assessments were conducted at the beginning and end of the trial, including questionnaire evaluations, and serum and fecal sample collection. Throughout the trial, participants in the placebo control group were required to take 1 sachet of placebo (maltodextrin) daily, while those in the probiotic CCFM6432 intervention group were instructed to take 1 sachet of CCFM6432 freeze-dried bacterial powder daily (with a viable count of ≥ 10^9^ CFU/d). See Figure 1 for the specific trial flowchart.

### Scale analysis

The Self-Rating Anxiety Scale (SAS), developed by Zung in 1971, has been clinically used for over fifty years^[21]^. This scale assesses the severity of anxiety symptoms and tracks changes in patients’ emotions during treatment. The HADS, developed by Zigmond AS and Snaith RP in 1983, is primarily used to assess anxiety and depression in patients in general hospitals^[22]^. HADS is a self-report scale consisting of two subscales, HADS-Anxiety and HADS-Depression, each containing seven items, with half of the items assessing anxiety and the other half assessing depression. This study simultaneously used both SAS and HADS to comprehensively evaluate the emotional status of patients, eliminating biases that may arise from the different focuses of the scales.

The Pittsburgh Sleep Quality Index (PSQI) is widely used for clinical assessment of sleep quality in patients with sleep disorders and mental disorders. Developed by Dr. Buysse *et al.* in 1989, the PSQI comprises 19 items used to evaluate an individual’s sleep quality over the past four weeks^[23]^. The PSQI has a sensitivity of 89.6%, a specificity of 86.5%, and a cutoff score of 5, with higher scores indicating poorer sleep quality.

The Gastrointestinal Symptom Rating Scale (GSRS), developed by Svedlund *et al.*, was used for this purpose^[24]^. The scale items were classified into “Bowel dysfunction syndrome”, “Indigestion syndrome”, “Dyspeptic syndrome”, and “Abdominal pain syndrome”. GSRS scores were generated from self-evaluation based on experiences during the past week.

### Fecal 16S rDNA amplicon sequencing analysis

The genomic DNA of the samples was PCR-amplified using primers targeting the V3-V4 region of bacterial 16S rDNA (341F: CCTAYGGGRBGCASCAG, 806R: GGACTACNNGGGTATCTAAT, with a 7-base barcode appended to the 5’ end of 341F to distinguish different samples). The PCR reaction system (50 µL) comprised Taq Plus master mix (Jiangsu Kangwei Century Biotechnology Co., Ltd.), primers (0.8 µM each), template DNA (2 µL), and nuclease-free water (added to make up the reaction volume to 50 µL). The PCR program included Step 1: 95 °C (3 min); Step 2: 95 °C (30 s), 52 °C (30 s), 72 °C (30 s), for 30 cycles; Step 3: 72 °C (6 min), 4 °C (4 min). Following PCR amplification, the products were subjected to 1.5% agarose gel electrophoresis (100 V, 100 mA, 30 min), and the bands of interest were purified to obtain the genomic DNA samples of intestinal bacteria from mice. The purity and concentration of the samples were assessed using Nanodrop, and a 50 µL mixed library with equal concentrations of all samples was constructed. Sequencing was performed using the Illumina MiSeq platform (Illumina, USA).

### Fecal untargeted metabolomics analysis

Accurately weigh 30-50 mg of vacuum freeze-dried fecal samples, and add 1 mL of methanol-water solution (methanol: water, 9:1, v/v) for homogenization. Incubate the mixture at -20 °C for 1 h to precipitate proteins, then centrifuge the mixture (15,000 *g*, 4 °C, 15 min). Concentrate the supernatant to dryness under vacuum, resuspend in 100 μL of methanol-water solution, and then centrifuge. The supernatant is filtered through a 0.22 μm organic filter membrane and subjected to liquid chromatography-mass spectrometry (LC-MS) analysis. Statistical analysis of the data was performed using MetaboAnalyst 5.0 software (http://www.metaboanalyst.ca/).

### Fecal lactic acid content analysis

Fecal samples from the population were freeze-dried to constant weight. Equal portions of 50 mg freeze-dried samples were transferred to 10 mL centrifuge tubes, and 300 μL of distilled water was added. After thorough mixing, homogenization was performed using a homogenizer. Subsequently, 1.2 mL of 10% chloroform was added to precipitate proteins. After centrifugation for 15 min (15,000 *g*, 4 °C), the supernatant was aspirated using a disposable sterile syringe, filtered through a 0.22 μm organic filter membrane, and transferred to 1.50 mL sample bottles for analysis using LC-MS. The concentration of metabolites was quantified based on a standard curve.

### Statistical analysis

Numeric results were presented as “mean ± standard deviation”. Data analysis and graph plotting were performed using Prism 9.0. Differences before (pre) and after (post) placebo or probiotic intervention were analyzed using paired *t*-tests. Comparisons of score changes between the probiotic and placebo groups were conducted using unpaired *t*-tests. *P*-values for multiple comparisons were adjusted using family-wise significance, and statistical significance was considered at a 95% confidence interval with a *P* < 0.05 in all comparisons.

## RESULTS

### CCFM6432 improves mood, sleep, and gastrointestinal function in patients with depression

**Figure 2:**
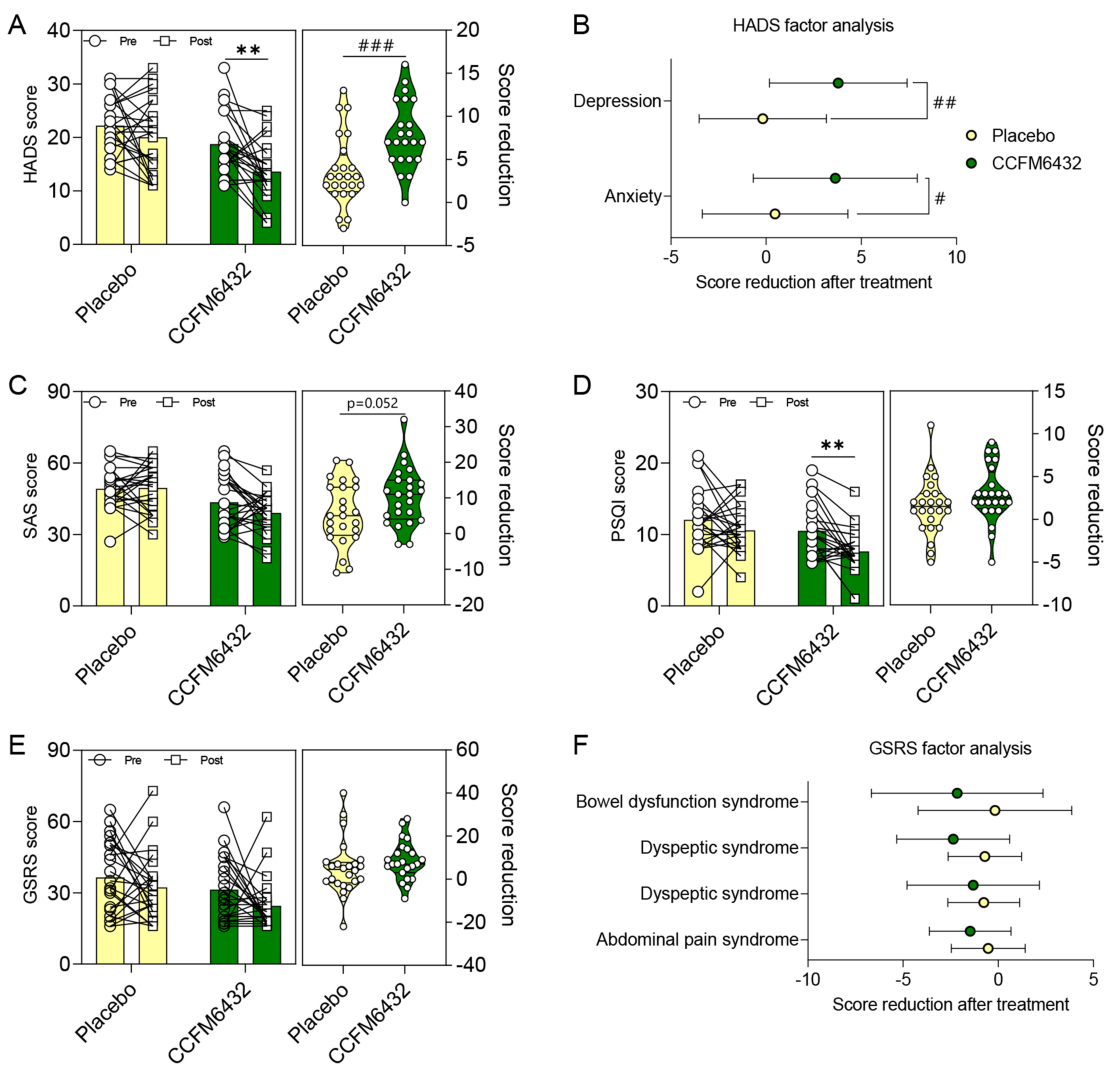
*Pediococcus acidilactici* CCFM6432’s effect on alleviating depression symptoms. (A) Score change of the HADS; (B) Analysis of HADS subscales for depression and anxiety; (C) Score change of the SAS; (D) Score change of the PSQI; (E) Score change of the GSRS; (F) Factor analysis of the GSRS. Colors distinguish groups. ^**^P < 0.01 in paired *t*-test. ^#^*P* < 0.05, ^##^*P* < 0.01, ^###^*P* < 0.001 in the unpaired *t*-tests. HADS: Hospital Anxiety and Depression Scale; SAS: Self-Rating Anxiety Scale; PSQI: Pittsburgh Sleep Quality Index; GSRS: Gastrointestinal Symptom Rating Scale.

Under ethical requirements, all patients continued to take antidepressant medications without interruption during the trial (see Table 1 for medication and demographic information). Therefore, to assess the effect of the probiotics, we analyzed the inter-group differences in the changes in scores before and after treatment, rather than focusing solely on within-group changes in scores before and after treatment. The results showed that the HADS score in the CCFM6432 group was significantly lower after treatment than before (*P* = 0.001), while there was no significant change in the HADS score in the placebo group before and after treatment (*P* = 0.174; Figure 2A). Moreover, compared to the placebo group, the CCFM6432 group showed a greater decrease in scores after treatment (*P* < 0.001; Figure 2A). Additionally, in terms of the “Anxiety” or “Depression” subscale scores, CCFM6432 showed a better effect on reducing the scores (*P* = 0.03 and *P* = 0.005, respectively; Figure 2B). Analysis of the SAS scores showed that there was no significant change in scores before and after treatment in the placebo group (*P* = 0.856 and *P* = 0.071, respectively), but the decrease in scores was greater in the CCFM6432 group (*P* = 0.052; Figure 2C).

**Table 1:** Medication and demographic information

**Variable**		**CCFM6432 (*n* = 20)**	**Placebo (*n* = 19)**	**Statistic value**	***P* value**
Age, Mean ± SD		37.40 ± 14.31	41.79 ± 17.81	1.554	0.416^a^
Sex, *n* (%)	Male	7	5	0.345	0.557^b^
	Female	13	14		
Race, *n* (%)	Chinese, Han nationality	20 (100)	19 (100)	N/A	N/A
BMI	Too light	1 (5)	3 (15)	1.284	0.45^a^
	Normal	13 (68)	10 (50)		
	Overweight	5 (27)	7 (35)		
Medication type, *n* (%)	Mirtazapine	2 (10)	3 (16)	0.292	0.589^b^
	Citalopram	1 (5)	4 (21)	2.246	0.134^b^
	Lorazepam	3 (15)	5 (26)	0.765	0.381^b^
	Zopiclone	0 (0)	1 (5)	1.08	0.298^b^
	Oxazepam	2 (10)	1 (5)	0.308	0.579^b^
	Venlafaxine	1 (5)	1 (5)	0.001	0.970^b^
	Sertraline hydrochloride	0 (0)	2 (11)	2.219,	0.136^b^
	Zolpidem tartrate tablets	1 (5)	1 (5)	0.001	0.970^b^
	Duloxetine	1 (5)	1 (5)	0.001	0.970^b^
	Tandospirone	2 (10)	1 (5)	0.308	0.579^b^
	Paroxetine	0 (0)	1 (5)	1.08	0.299^b^
	Olanzapine	1 (5)	1 (5)	0.001	0.970^b^
	Fluvoxamine maleate tablets	0 (0)	2 (11)	2.219	0.136^b^
	Aripiprazole	1 (5)	1 (5)	0.001	0.970^b^
	Quetiapine	2 (10)	0 (0)	2.003	0.157^b^
	Clonazepam	1 (5)	0 (0)	0.975	0.323^b^
	Unknown	4 (25)	4 (21)	0.007	0.935^b^
Smokers, *n* (%)	Smoker	3 (15)	5 (26)	0.765	0.381^b^

a Statistical analysis by *t*-test. ^b^Statistical analysis by chi-square test. n: Number of participants; N/A: not available. BMI: body mass index.

Patients with depression often experience sleep and gastrointestinal issues throughout the course of the disease, with reported comorbidity rates as high as 90% for sleep disorders and about 60% for gastrointestinal dysfunction^[25,26]^. We evaluated the sleep quality and gastrointestinal function of patients using the PSQI and GSRS scales, respectively. Unlike the placebo (*P* = 0.156), only CCFM6432 treatment significantly reduced PSQI scores (*P* = 0.001); however, there was no significant difference in score changes before and after treatment between the two groups (*P* = 0.149; Figure 2D). Both placebo and CCFM6432 had no significant effect on patient gastrointestinal function [Figure 2E and F].

### CCFM6432 treatment does not significantly disturb the microbial structure of patients

**Figure 3:**
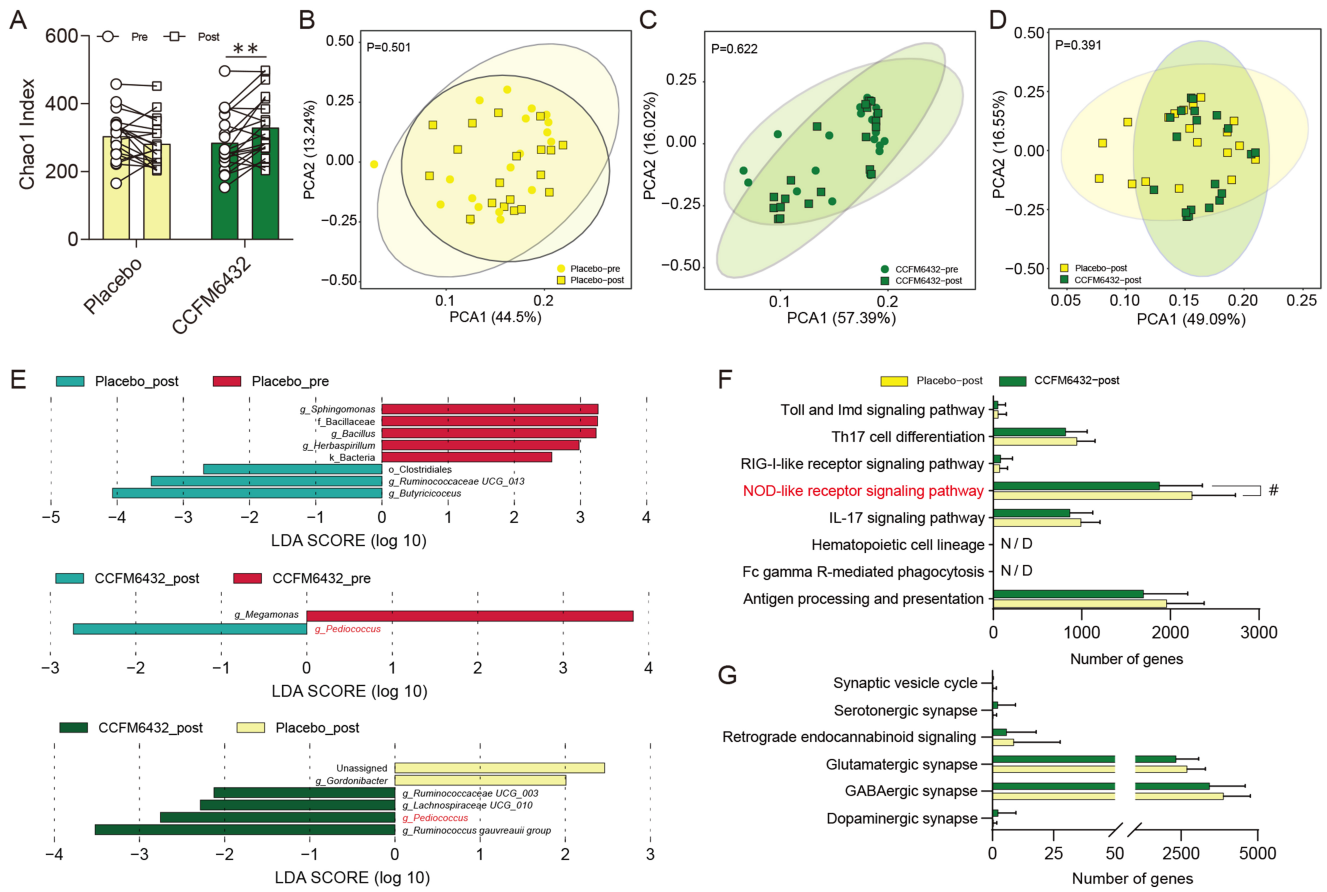
*Pediococcus acidilactici* CCFM6432’s effect on the gut microbiota of depressed patients. (A) α-diversity - Chao1 index; ^**^P < 0.01 in paired *t*-test; (B-D) PCA of gut microbiota composition; PERMANOVA was used to assess β-diversity differences between groups; (E) LEfSe analysis of differential microbial species between groups (Wilcoxon rank-sum test, α < 0.05 and log LDA > 2.0 were used as the threshold); (F and G) Predicted genomic functions of gut microbiota and enrichment analysis of immune and neural function-related signaling pathways. The metagenomic composition was predicted from 16S rRNA sequences and annotated based on the reference database (KEGG). ^#^*P* < 0.05 in the unpaired *t*-tests. PCA: Principal component analysis; PERMANOVA: permutational multivariate analysis of variance; LEfSe: linear discriminant analysis effect size; LDA: linear discriminant analysis; KEGG: Kyoto Encyclopedia of Genes and Genomes.

Following intervention with *Pediococcus acidilactici* CCFM6432, the Chao1 index significantly increased (*P* = 0.004, Figure 3A), indicating a significant increase in species richness of the gut microbiota in patients with depression. However, CCFM6432 did not significantly affect the beta diversity of the microbiota [Figure 3B-D], indicating minimal disturbance to the composition of the gut microbiota. Further identification of inter-group differences in specific microbial species was conducted through linear discriminant analysis effect size (LEFSe) analysis based on the Kruskal-Wallis rank sum test. The major contributing species to differences in the gut microbiota before and after CCFM6432 treatment were *Megamonas* and *Pediococcus* [Figure 3E]. Previous studies have shown associations between *Megamonas* and metabolic diseases such as alcoholic fatty liver and diabetes^[27,28]^. Additionally, the abundance of *Megamonas* in the feces of autistic children was significantly higher than in healthy children^[29]^. In contrast, differences in microbial species after placebo treatment were mainly contributed by *Pediococcus*, *Ruminococcaceae UCG_003*, *Lachnospiraceae UCG_010*, and the *Ruminococcus gauvreauii group*. Previous research has indicated that the abundance of Lachnospiraceae, Ruminococcaceae, and *Ruminococcus* in the gut of patients with severe depression is lower than in healthy controls^[30,31]^, suggesting that taking CCFM6432 helps promote the proliferation of microbial species positively associated with host emotional responses.

Considering the well-known ability of *Pediococcus* to produce lactic acid and inhibit the growth of pathogenic bacteria, we performed annotation analysis of the microbial genome functions (focusing on immune and nervous system-related signaling pathways) to explore the potential impact of the gut microbiota on host physiology. We found that the annotated genes in the pathways of Antigen processing and presentation, Th17 cell differentiation, and NOD-like receptor signaling pathway were lower in the CCFM6432-modified microbiota [Figure 3F]. Particularly, the NOD-like receptor signaling pathway, a critical pathway involved in pathogen recognition and innate immune response, showed a statistically significant difference between the CCFM6432 and placebo groups (*P* = 0.020). However, there were no significant inter-group differences in neural system-related signaling pathways [Figure 3G].

### Positive correlation between increased intestinal lactic acid levels mediated by probiotics and improvement in patient mood

Considering the alterations in specific gut microbial taxa abundance in patients with depression following probiotic intervention, it was hypothesized that this might lead to significant changes in the spectrum of intestinal metabolites. To investigate this, a non-targeted metabolomic approach based on LC-MS was employed to detect the metabolite composition of fecal samples from patients with depression. The obtained data were compared and annotated against the Mzcloud and Kyoto Encyclopedia of Genes and Genomes (KEGG) databases, with substances matched with a score greater than 80 being selected. Ultimately, 137 substances were identified in positive ion mode and 257 substances in negative ion mode.

**Figure 4:**
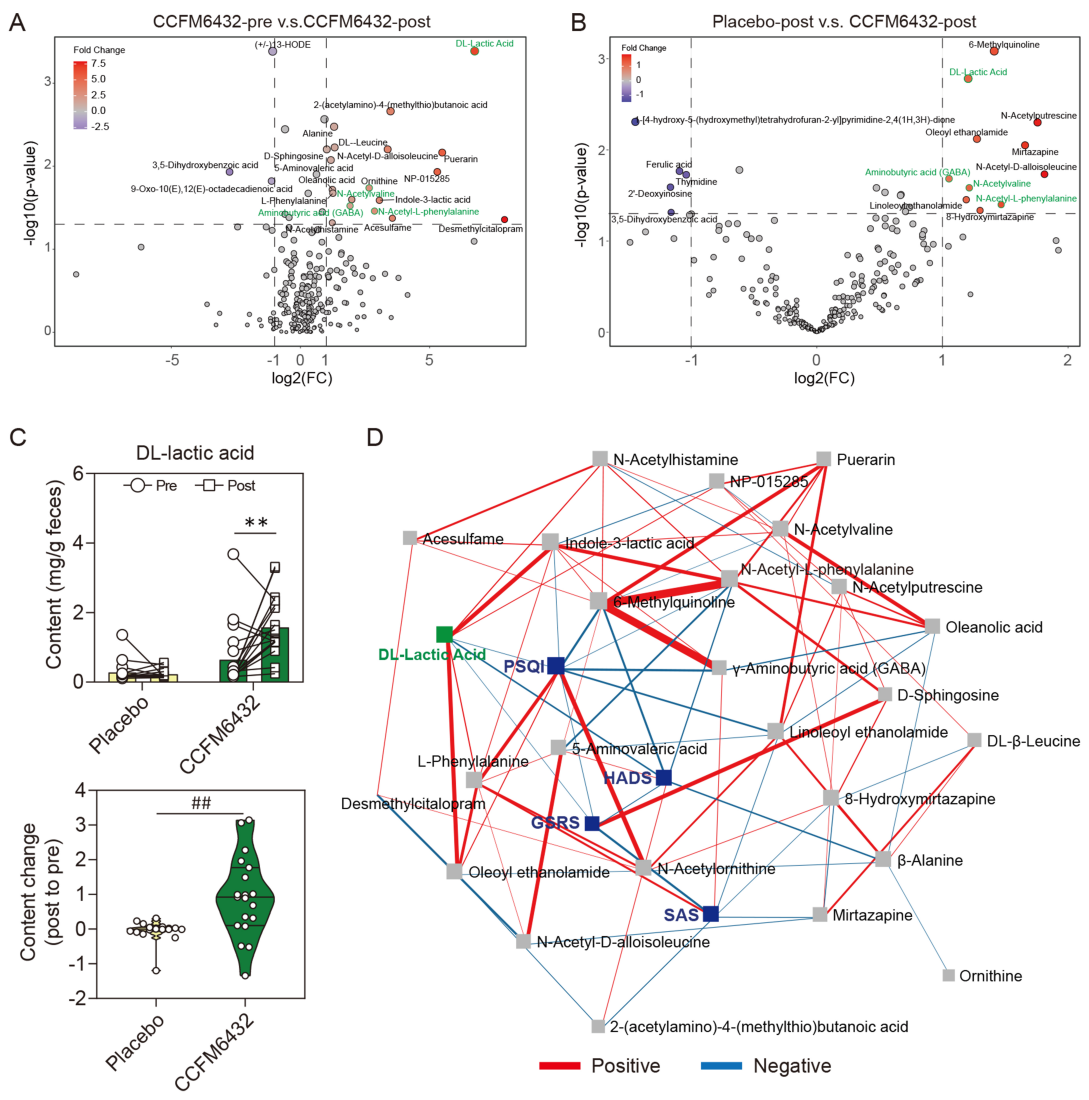
*Pediococcus acidilactici* CCFM6432’s effect on the fecal metabolome. (A) Differences in gut microbiota metabolites before and after intervention in the CCFM6432 group. Fold change > 2 and *P* < 0.05 were set as the thresholds for biomarker screening; (B) Differences in gut microbiota metabolites between the placebo group and the CCFM6432 intervention group at the end of treatment. Fold change > 2 and *P* < 0.05 were set as the thresholds for biomarker screening; (C) Absolute quantification of DL-lactic acid. ^**^P < 0.01 in paired *t*-test. ^##^*P* < 0.01 in the unpaired *t*-tests; (D) Correlation analysis between changes in scale scores and changes in gut metabolites before and after intervention. The correlation was established by DSPC network. The size of each node is proportional to the number of connections. Red lines represent a positive correlation, and blue lines represent a negative correlation. The width of the line represents the correlation strength. DSPC: Debiased sparse partial correlation.

To further explore key biomarkers before and after CCFM6432 intervention, a volcano plot was used to display metabolites with fold change values greater than 2 and p-values less than 0.05 (*t*-test) between groups. Compared to pre-CCFM6432 intervention or post-placebo intervention, the levels of DL-lactic acid, GABA, N-acetyl-L-phenylalanine, and N-acetylvaline in the intestine were significantly elevated after CCFM6432 intervention [Figure 4A and B]. Among these substances, lactic acid exhibited the most significant inter-group differences. Furthermore, absolute quantification of lactic acid in feces using LC-MS validation confirmed that the intake of CCFM6432 significantly increased the level of lactic acid in the intestines of patients with depression, with a significantly higher increase than the difference before and after placebo treatment [Figure 4C]. Finally, for the differential metabolites identified in Figure 4A and B, debias sparse partial correlation (DSPC) network analysis was conducted to assess the correlation between changes in metabolite levels and changes in mood, sleep, and gastrointestinal scale scores [Figure 4D]. It was found that there was a significant negative correlation between the change in fecal lactic acid content and the change in HADS score (i.e., higher lactic acid levels were associated with lower HADS scores; Partial Coeff. = -1, *P* = 0.024); however, the change in lactic acid levels was negatively correlated with changes in PSQI and GSRS scores, but without statistical significance (Partial Coeff. = -1, *P* = 0.154; Partial Coeff. = -0.363, *P* = 0.179, respectively). Additionally, a significant negative correlation was observed between the change in N-Acetyl-L-phenylalanine content and the change in HADS score (Partial Coeff. = -1, *P* = 0.046).

## DISCUSSION

In this study, we evaluated the effects of *Pediococcus acidilactici* CCFM6432 on emotional performance, gastrointestinal function, gut microbiota, and the distribution of their metabolic products in patients with depression. Although the intake of this strain did not significantly alter the overall structure of the gut microbiota (beta diversity) in patients, *Pediococcus acidilactici* CCFM6432 demonstrated good colonization characteristics, becoming a distinguishing species between the gut microbiota of the two groups. Notably, it significantly increased the lactic acid content in the gut, and this change was positively correlated with improvements in mood, gastrointestinal function, and sleep quality. Specifically, we observed a significant reduction in the annotation abundance of the NOD-like receptor signaling pathway in the gut microbiome. Given the well-known antibacterial effects of lactic acid, the brain-beneficial functions of *Pediococcus acidilactici* may be associated with the inhibition of inflammation signaling pathways mediated by microorganisms.

Over the years, the role of the immune system has been closely linked to the etiology and pathophysiology of depression^[32]^. For instance, clinical studies have found significantly elevated levels of pro-inflammatory cytokines, such as interleukin-6 (IL-6), TNF-α, and C-reactive protein, in the serum or cerebrospinal fluid of depressed patients^[33,34]^. Patients undergoing cytokine therapy for viral infections or tumors often experience severe depressive symptoms, accompanied by an increased risk of suicide^[35]^. Notably, disruptions in gut microbiota may be an important risk factor for inflammatory depression, as the gut hosts numerous immune cells, such as macrophages and dendritic cells. Moreover, intestinal epithelial cells express a variety of pattern recognition receptors, including Toll-like receptors (TLRs) and NOD-like receptors, which can be activated by specific metabolites and bacterial cell wall components such as lipopolysaccharides (LPS), peptidoglycans, and lipoteichoic acids, subsequently activating the immune system^[36]^. This activation initiates innate and adaptive immune responses as well as inflammation^[37,38]^. A recent study found that compared to non-inflammatory depression or control groups, patients with inflammatory depression exhibit an increase in the abundance of *Proteobacteria* and a decrease in butyrate-producing bacteria like *Faecalibacterium* in their gut microbiota^[39]^. Transplanting fecal microbiota from individuals with inflammatory depression into mice has been shown to induce depressive and anxiety-like behaviors, accompanied by increased peripheral and central inflammatory markers and intestinal permeability. Mechanistically, the TLR-4/NF-κB and NLRP3 inflammasome signaling pathways partially mediate this interaction, highlighting the intricate relationship between gut microbiota, inflammation, and mood disorders^[39]^.

In previous research, we found that administering CCFM6432 could reduce the abundance of pathogenic microorganisms, such as *Escherichia-shigella*, in the guts of chronically stressed mice^[40]^. *In vitro* antibacterial tests revealed that this strain inhibits the growth of *Escherichia coli*, *Staphylococcus aureus* subsp. *aureus*, *Listeria monocytogenes*, and *Salmonella typhimurium* through the production of lactic acid rather than bacteriocins^[41]^. Additionally, in animal models, CCFM6432 administration helped reverse the abnormal increase in gut barrier permeability and serum LPS levels caused by chronic stress. This intervention further inhibited the expression of receptor proteins and pro-inflammatory cytokines in the TLR4/NF-κB signaling pathway in the brain, thereby reducing the overactivation of microglia in the hippocampus^[41]^. The observations from this human study are logically consistent with previous results, suggesting that CCFM6432 may alleviate immune activation associated with depression through lactic acid production, thereby improving the host’s emotional state. In contrast to the results observed in mice, the probiotic did not significantly alter the overall structure of the gut microbiota in the human cohort. This outcome aligns with the intrinsic definition of probiotics, which emphasizes their gentle and non-invasive mode of action. Many classic probiotics have been isolated from the early-life gut microbiota of infants, indirectly highlighting their low immunogenicity and minimal host stimulation. Moreover, there has been no consistent evidence to suggest that gut microbiota diversity is significantly altered in patients with depression, nor that such alterations are a direct consequence of the disorder^[42]^. Therefore, the theoretical basis for developing probiotic therapies aimed at improving gut microbiota structure remains insufficiently substantiated. This further underscores the need for future development of psychiatric probiotics to focus more on the effective substances that mediate their therapeutic effects.

Clinical depression patients often experience various gastrointestinal dysfunctions and sleep disorders^[10]^. Our research has shown that intervention with *Pediococcus acidilactici* CCFM6432 significantly alleviated these symptoms, which aligns with clinical findings of other psychobiotics^[43,44]^. However, unlike most probiotics, the intake of this strain did not significantly alter the overall structure of the patients’ gut microbiota. Its mechanism may involve increasing the levels of its metabolic product, lactic acid, in the gut, reducing the abundance of pathogenic bacteria and associated inflammation, thereby improving depressive symptoms. Nevertheless, the specific interactions between gut metabolites (such as lactic acid), circulating inflammatory factors, and brain immune status require further investigation.

There are, however, some limitations in our study that future research should address. Firstly, due to the small sample size and short intervention period, we cannot resolve the individual differences in symptoms and responses to probiotic intervention caused by different living environments, genetic backgrounds, and medication interventions in depressed patients. Future studies should increase sample sizes and intervention durations, and controlling or stratifying participants’ diets, medications, and living environments may help reduce external variables. Secondly, our hypothesis that *Pediococcus acidilactici* CCFM6432 alleviates depressive symptoms by reducing systemic inflammation is based on functional predictions of the gut microbiota and preclinical results. Further research is needed to provide evidence on the specific interactions between gut metabolites (such as lactic acid), circulating inflammatory factors, and brain immune status. Additionally, the measurement of lactic acid in this study was conducted using a non-targeted metabolomics approach, without distinguishing between D- and L-lactic acid. Future studies should aim to identify the specific molecular configuration of lactic acid responsible for the observed effects.

In conclusion, the utilization of probiotics for mitigating mood disorders holds considerable potential. This study is the first to report the clinical efficacy of a single strain of *Pediococcus acidilactici* in the adjunctive treatment of depression and its regulatory effects on the gut microbiota of patients. Future investigations should prioritize exploring optimal intervention dosages and elucidating the pharmacological mechanisms of probiotics, aiming to contribute to the advancement of innovative antidepressant strategies.

## DECLARATIONS

### Availability of data and materials

All data are available in the main text. Other raw data that support the findings of this study are available from the corresponding author upon reasonable request.

### Copyright

© The Author(s) 2024.
